# Complete genome sequence of *Deinococcus sonorensis* type strain KR-87: a rare biofilm-producing representative of the genus *Deinococcus*

**DOI:** 10.1128/mra.00732-24

**Published:** 2024-09-16

**Authors:** Leilani S. Boren, Ryan A. Grosso, Alex N. Hugenberg-Cox, Jonathan T.E. Hill, Chad M. Albert, James M. Tuohy

**Affiliations:** 1 Biology Department, Glendale Community College, Glendale, Arizona, USA; University of Southern California, Los Angeles, California, USA

**Keywords:** *Deinococcus*, Oxford Nanopore, biofilms, genomics, extremophiles

## Abstract

Here, we present a complete genome of *Deinococcus sonorensis* KR-87^T^, a biofilm-producing mesophile from the Arizonan Sonoran Desert. The sequence, assembled using Oxford Nanopore Technologies’ long-read sequencing platform, predicts a genome size of 4.78 Mbp, with 6 replicons, 4,361 protein-coding genes, and a G+C content of 69.0%.

## ANNOUNCEMENT

The genus *Deinococcus* (1) is uniquely resistant to DNA-damaging agents such as ionizing and non-ionizing radiation and oxidative stress (2) and is made up of at least 93 named species (https://www.bacterio.net/genus/deinococcus). *Deinococcus sonorensis* KR-87^T^ is a radioresistant extremophile, which produces a robust biofilm unlike any described in the genus (3). This report provides a genomic foundation on which to study the environmental and comparative physiology of this unique *Deinococcal* species.

*D. sonorensis* KR-87^T^ was originally isolated by Rainey and colleagues (3) from the top 2 cm of arid soil in a sparsely vegetated region of the Sonoran Desert between Phoenix and Tucson, Arizona. It is a Gram-positive, nonmotile, non-spore-forming, spherical or short rod-shaped cell. A freeze-dried sample of *D. sonorensis* KR-87^T^ (DSM 21209) was obtained from the German Collection of Microorganisms and Cell Cultures (Leibniz Institute, DSMZ) and grown in Reasoner’s 2A agar medium for 48 hours at 30℃. Multiple colonies were transferred to Reasoner’s 2B liquid medium, vortexed, and briefly pelleted before being resuspended in lysis buffer (DNeasy Ultraclean Microbial Kit, Qiagen). Cells were lysed using a Bead Mill 4 bead-beater (Fisher Scientific, USA) for 60 s at 4 m/s. DNA extraction was performed using the DNeasy Ultraclean Microbial Kit (Qiagen, Germany) and Quick-DNA Fungal/Bacterial Miniprep Kit (Zymo, USA) and further purified with the Monarch PCR & DNA Cleanup Kit (New England BioLabs, USA). DNA concentration and quality were determined using the NanoDrop One (Thermo Fisher, USA) and the Qubit 4.0 fluorometer with the Qubit 1× dsDNA HS Kit (Thermo Fisher, USA).

Nanopore sequencing (Oxford Nanopore Technologies) was accomplished using a Ligation Sequencing Kit V14 (SQK-LSK114) with no shearing or size selection. The sequencing library was loaded onto a R10.4.1 FLO-MIN114 flow cell on a MinION Mk1B sequencer. MinKNOW v24.02.8 (ONT) was used for sequence planning and data acquisition, with the super-accurate basecalling mode enabled. Dorado v7.3.11 (4) was used as the basecalling algorithm.

The Galaxy bioinformatics platform (https://usegalaxy.org.au) (5) facilitated raw sequence processing and annotation, and FastQC v0.74 + galaxy0 (6) provided quality control for preprocessed sequence data. Flye v2.9.3+galaxy0 (7) was used for *de novo* genome assembly with base parameter settings, and the quality of the genome was verified with QUAST v5.2.0 (8). There was a total of 432,770 reads with an *N*_50_ of 8.85 kb and 342× coverage. Annotation was provided by NCBI’s Prokaryotic Genome Annotation Pipeline v6.7 (9).

The completed genome assembly was found to be 4,785,116 bp in total aligned length with an *N*_50_ of 3,027,404 bp, 98.48% complete across 6 circular replicons (Table 1). There are a predicted 4,361 protein coding sequences (CDS), 58 tRNA genes, 9 rRNA genes, 9 ncRNAs, 47 pseudogenes, and an overall G + C content of 69.0%. Genome completeness was assessed by BUSCO v5.5.0 (10) and yielded a total BUSCO search of 124 (complete and single-copy BUSCO; 107). Average Nucleotide Identity (11) analysis indicated that *Deinococcus aquiradiocola* JCM 14371^T^ is the closest known relative to *D. sonorensis* KR87^T^ (82.73% match).

**TABLE 1:** Replicon characteristics of *D. sonorensis* KR-87T

Replicon	Length (bp)	CDS count	tRNA count	G+C content (%)	Accession no.
Chromosome	3,027,404	2,905	52	69	NZ_CP158299.1
pDson01	484,959	382	0	69.5	NZ_CP158297.1
pDson02	449,785	368	2	68.5	NZ_CP158300.1
pDson03	437,185	379	4	69.5	NZ_CP158298.1
pDson04	309,287	259	0	69	NZ_CP158296.1
pDson05	76,496	68	0	63	NZ_CP158301.1
Total	4,785,116	4,361	58		

## Data Availability

The raw data, found under BioProject accession number, PRJNA1121405, were submitted to the NCBI Sequence Read Archive (SRA) under accession number SRX24845611 (Nanopore reads in fastq format).
